# Genetic background influences the effect of thirdhand smoke exposure on anxiety and memory in Collaborative Cross mice

**DOI:** 10.1038/s41598-021-92702-1

**Published:** 2021-06-24

**Authors:** Li He, Pin Wang, Suzyann F. Schick, Abel Huang, Peyton Jacob, Xu Yang, Yankai Xia, Antoine M. Snijders, Jian-Hua Mao, Hang Chang, Bo Hang

**Affiliations:** 1 Department of Internal Hematology, Zhongnan Hospital of Wuhan University, Wuhan, 430071 Hubei China; 2 Department of Gastroenterology, Nanjing Drum Tower Hospital, Affiliated Hospital of Nanjing University Medical School, Nanjing, 210008 Jiangsu China; 3 Division of Occupational and Environmental Medicine, Department of Medicine, University of California, San Francisco, CA 94143 USA; 4 Division of Cardiology, Department of Medicine, Clinical Pharmacology Program, University of California, San Francisco, CA 94143 USA; 5 School of Public Health, Nanjing Medical University, Nanjing, 211166 Jiangsu China; 6 Biological Systems and Engineering Division, Lawrence Berkeley National Laboratory, Berkeley, CA 94720 USA

**Keywords:** Genetics, Neuroscience, Risk factors

## Abstract

Growing evidence indicates that thirdhand smoke (THS) exposure induces many adverse health effects. However, it is unclear how THS exposure affects behavior and how host genetic background modulates phenotypic changes. Here we used the Collaborative Cross (CC) mouse population-based model to assess behavioral alterations immediately after THS exposure from 4 to 9 weeks of age. We first measured anxiety-like behavior in six strains using light/dark box combined with a custom multivariate mouse tracking system. We developed an anxiety risk scoring system based on anxiety-related traits and then evaluated the THS impact on them. THS exposure significantly decreased anxiety risk in CC019 (*P* = 0.002) and CC051 (*P* = 0.009), but increased anxiety risk in CC036 (*P* < 0.001), while the other three strains did not show significant changes in anxiety-related traits. Such differences were driven by female mice for the six measures of anxiety-like behavior. Memory potential was measured in the same cohort of mice using the passive avoidance assay. Both THS-exposed male and female CC019 mice displayed significant memory loss compared to controls while no significant changes were found in the other five strains. This study provides strong evidence that THS exposure leads to strain-dependent changes in anxiety-like behavior and memory, suggesting that host genetic variations play a critical role in individual susceptibility to THS-induced effects.

## Introduction

Thirdhand smoke (THS) has gained widespread attention in the last decade as a previously overlooked source of exposure to toxins in indoor smoking environments. THS is defined as residual tobacco smoke adsorbed onto indoor surfaces and in dust after active smoking has ceased, some of the adsorbed constituents can be re-emitted into the gas-phase and/or react with other environmental pollutants to form more hazardous compounds^1,2^. Although secondhand smoke (SHS) dissipates in hours, THS is more persistent, ranging from days to months, even years, after smoking has ceased. Non-smokers can be exposed to THS through several routes including the involuntary inhalation, ingestion, and dermal uptake of indoor pollutants. Small children may gain more exposure than adults because their age-related behaviors bring them in close contact with surfaces and dust contaminated with THS toxins. In addition, their physiological characteristics such as lower capacity to metabolize the toxins and high toxins/body weight ratio potentially make them more sensitive to adverse health effects associated with THS exposure.

Nicotine is a chemical in tobacco and causes addiction. It is the most prevalent constituent in THS^1,2^. In addition, there are more than 100 chemical compounds that have been identified thus far in airborne or surface THS^2–4^, including products formed de novo from the reaction of nicotine with environmental pollutants such as nitrous acid^5^ and ozone^6,7^. There is evidence that children have higher levels of THS constituents in urine such as nicotine and 4-(methylnitrosamino)-1-(3-pyridyl)-1-butanol (NNAL), a metabolite of 4-(methylnitrosamino)-1-(3-pyridyl)-1-butanone (NNK) in homes of parents who smoked, or even never smoked inside their home, than the children of nonsmokers^8,9^. Therefore, THS pollution represents a new challenge for public health, particularly for small children who may represent a particularly exposed group.

The toxic compounds in THS may cause adverse biological and health effects in exposed individuals. Studies have shown that exposure to THS in vitro causes a variety of molecular and cellular changes at environmentally relevant doses^10–14^ and that in mouse models THS exposure impairs multiple organs, affects immune parameters, and induces lung cancer, especially when exposure occurred at early age^11,15–17^. Exposure to environmental toxins is a risk factor for human brain health, including memory, cognition, anxiety, and even neurological disease^18–20^. Many in vitro and animal studies have demonstrated associations between exposure to indoor environmental pollutants and behavioral alterations^21^. However, the only evidence for THS exposure effects on behavior was reported in one study in 2014, which described hyperactivity in THS-exposed C57BL/6 mice using the open field test^15^. Together, these observations warrant further investigations into the effects of THS exposure on other behavioral endpoints.

There is a wide variation in the animal and human response to ubiquitous environmental pollutants^22^. Interestingly, we previously observed an increase in lung cancer incidence in THS-treated A/J mice^11,23^ but not in similarly treated C57BL/6 mice (ref 17, and Martins-Green, personal communication). In general, there is a critical knowledge gap in our understanding of the genetic basis for interindividual responses to THS exposure. Studies in animal model systems can help fill this gap by determining the genetic basis for the interindividual response to THS using well-controlled, genetically defined population-based model systems. In recent years, we have used the genetically diverse Collaborative Cross (CC) mouse system, which contains a level of genetic and phenotypic diversity comparable to the human population, to study linkage of genetic variants to phenotypic alterations, such as motor deficits^24^, memory performance^25,26^, anxiety-like behavior^27^, gut microbiome composition^28^, and cancer susceptibility^29^.

The aim of this work was to investigate how host genetic background influences the effect of THS exposure on behavior endpoints. We chose two widely used methods for assessing anxiety-like behavior and memory across six CC mouse strains selected to cover the wide range of anxiety-like and memory phenotypes observed in the CC mice. This study provides new evidence on the effect of THS exposure on behavior and emphasizes the complex interaction between host genetics and environmental THS exposure on health.

## Methods

### Ethics statement

All animal experiments were performed at the Lawrence Berkeley National Laboratory (LBNL) and the study was carried out in strict accordance with the Guide for the Care and Use of Laboratory Animals of the National Institutes of Health (NIH). The animal use protocol was approved by the Animal Welfare and Research Committee of LBNL (Protocol File Number 270024). The study was carried out in compliance with the ARRIVE guidelines. Euthanasia of animals is by CO_2_ exposure followed by cervical dislocation in compliance with the AVMA guidelines for the euthanasia of animals (2020).

### THS sample preparation and chemical characterization

A controlled laboratory system located at University of California at San Francisco (UCSF) was used to generate THS samples on cotton terry cloth, as previously described^10,11^. The cloth substrates were used as surrogates for indoor surfaces, onto which SHS chemicals could adsorb. Briefly, clean 100% cotton terrycloth samples were repeatedly exposed to SHS in a 6-m^3^ stainless steel chamber over 520 days. During smoking, a total of 2804 mg of total particulate material was introduced into the steel chamber. This is equivalent to the smoke from 200 to 280 cigarettes over 520 days, or approximately half a cigarette per day. THS cloth was removed from the smoke, vacuum-packed in Mylar film and stored at − 20 °C until use.

For chemical characterization of the samples, the extraction of compounds in THS-laden and control cotton cloth samples was performed with Dulbecco's Modified Eagle's Medium (DMEM) as previously described^10^. A collection of 6 targeted THS compounds in the DMEM samples was analyzed following the procedures described in the previous study^10^, using liquid chromatography–tandem mass spectrometry (LC–MS/MS) as previously described^30^. The concentrations for the THS compounds tested: nicotine: 1.172 ug/ml, NNK: 2.30 ng/ml, NNN: 0.57 ng/ml, NAT: 0.49 ng/ml, NAB: 0.24 ng/ml, and nicotelline: 11.28 ng/ml.

### CC mouse strains and THS exposure

220 mice from six CC strains, CC001/Unc, CC019/TauUnc, CC036/UncJ, CC037/TauUnc, CC042/GeniUnc and CC051/TauUnc, were used for this study. These six CC strains represent low, intermediate and high anxiety risk tiers based on the baseline level anxiety study across 30 CC strains described by Jin et al.^27^ and allow for observations of the potential effect of THS exposure on anxiety-like behavior without being constrained by a floor or ceiling effect of our measurements. More specifically, CC042 and CC019 were assigned low anxiety strains (LA), CC037 and CC036 were assigned intermediate anxiety strains, while CC001 and CC051 were assigned high anxiety (HA) strains^27^. All mice were purchased from the Systems Genetics Core Facility at the University of North Carolina (UNC). Genetic information of these 6 CC strains can be found on the UNC Systems Genetics Core website (http://csbio.unc.edu/CCstatus/index.py).

Mice were acclimated at the Lawrence Berkeley National Laboratory (LBNL) for 8 weeks prior to the initiation of breeding. All six strains were weaned at 21 days and raised in groups of 3–5 mice (same age and sex) per cage to allow social interaction. For CC019, mice diagnosed with congenital hydrocephalus were excluded from this study. Mice were fed a standard chow diet (PicoLab Rodent diet 5053), raised in individually ventilated cages in a light-controlled room with 12:12 h light/dark cycle.

For THS exposure, mice from each strain were randomly divided into exposed and unexposed (control) groups immediately after weaning. Mice were exposed to THS-exposed terry cloth or control cloth from 4 to 9 weeks of age as follows. A 3.4 g (10 × 10 cm^2^) swatch of THS-exposed cloth or control cloth was added to the standard bedding in the cages, and the cloth swatches were replaced once a week during the standard cage change. The nicotine loading of the swatches was 23.4 μg/g for a total of 79.56 μg. It should be noted that such a value is close to the ingestion exposure of a toddler, as estimated by Bahl et al.^31^.

### Light/dark box anxiety test and analysis

The anxiety-like behavior test was performed prior to the memory test to avoid the potentially confounding effect of the foot shock on the anxiety phenotype measurements. Both memory and anxiety behavior tests were conducted on all mice during the daytime between 1 and 4 p.m. The anxiety and memory tests were always run concurrently, with the anxiety test always run first followed by the memory test, and the average time between the two tests was 20–30 s.

To characterize the effects of the THS exposure on anxiety-like behavior, we first assessed anxiety 1 day after the end of 6-week THS exposure using the light/dark box (Stoelting Co., Wood Dale, IL)^32^. The apparatus has two compartments: a covered black compartment (20 cm × 40 cm × 35 cm, width × depth × height) and an open illuminated transparent compartment of the same size. A small opening connects the dark and light compartments. The lit compartment is illuminated from the overhead light. There dark compartment is covered by a black lid preventing overhead light from entering the dark compartment. The lighting levels for the lit and dark compartments were 845 and < 2 lx, respectively. A mouse was placed in the corner of the light box and allowed to explore the enclosure freely for 5 min. A video camera recorded the mouse movements in the light box. This test is based on the time the mouse spent in the light box, and its related exploratory behaviors, which are reliable parameters for assessing anxiety-related effects^33,34^. After the light/dark box test, we deployed a computational pipeline for multivariate characterization of mouse behavior by the following steps: (1) mouse tracking from video, (2) behavior profiling, and (3) multivariate phenotype extraction^27^. The first two steps resulted in the mouse behavior profile, which characterizes the dynamic mouse size in the light chamber across time for both perceptual quality control and phenotype definition. The last step produces six anxiety related phenotypes for further evaluation of the impact of THS on anxiety.

### Anxiety risk evaluation

To evaluate the impact of THS on mouse anxiety, we improved and extended our previous work^27^ to construct a multivariate anxiety risk scoring system as follows:

$$\begin{aligned} Anxiety\;Risk\;Score & = Intercept + ~\alpha _{1} \;NumberFullTransition + ~\alpha _{2} \;NumberPartialTransition \\ & \quad + ~\alpha _{3} \;LatencyTimeFirstTransition + ~\alpha _{4} \;TotalTimeInLight \\ & \quad + ~\alpha _{5} \;TravelingDistanceInLight + ~\alpha _{6} \;AverageSpeed~ \\ \end{aligned}$$

where six anxiety-like phenotypes significantly contribute to the risk scoring system were adopted. Specifically, the risk scoring system was constructed as a linear regression model (Pseudo-R^2^ (Cragg-Uhler) = 0.84) based on the anxiety-like phenotypes extracted from the light–dark experiments of 445 mice across 30 CC strains^27^, where the low anxiety group (LA) and high anxiety group (HA) discovered in^27^ were assigned an anxiety risk score (ARS) of 0 and 1, respectively. In addition, each anxiety-like phenotype during both model construction (i.e., on the training cohort^27^) and risk evaluation (i.e., on the THS cohort in this study) was independently normalized (i.e., z-score normalization) to accommodate potential batch effects.

### Passive avoidance memory test

To assess the effects of THS exposure on short-term memory, we performed the passive avoidance test 1 day after the end of the 6-week exposure using the Panlab passive avoidance box (Panlab, Harvard Apparatus, Holliston, Massachusetts. LE870/872). The apparatus is composed of a larger white compartment and a smaller black compartment connected by an automatic sliding door. During the training phase, a mouse was placed in the light compartment and the door was immediately opened for 30 s. When the mouse innately crossed to the dark compartment, it received a mild foot shock for 5 s (0.3 mA). The time a mouse spent in the light compartment before the first entry to the dark compartment was recorded. Three days after the training phase, the mouse was again placed in the light compartment and the passive avoidance response was evaluated by measuring the latency to enter the dark compartment. Only one mouse was placed in the room with the experimental apparatus at a time.

### Statistics

Statistical analysis was performed using IBM SPSS software (version 24). Since the behavior phenotypes are not normally distributed, we applied the non-parametric Mann–Whitney U test to compare the differences between the THS-exposed and control groups in memory, anxiety-like phenotypes and anxiety risk score, including sex differences for each of these categories. A *P* value less than 0.05 was used as the cut point for determining statistical significance. Linear regression model for anxiety risk scoring system was constructed in R (version 3.6.0) with stats package (version 3.6.0).

## Results

**Figure 1:**
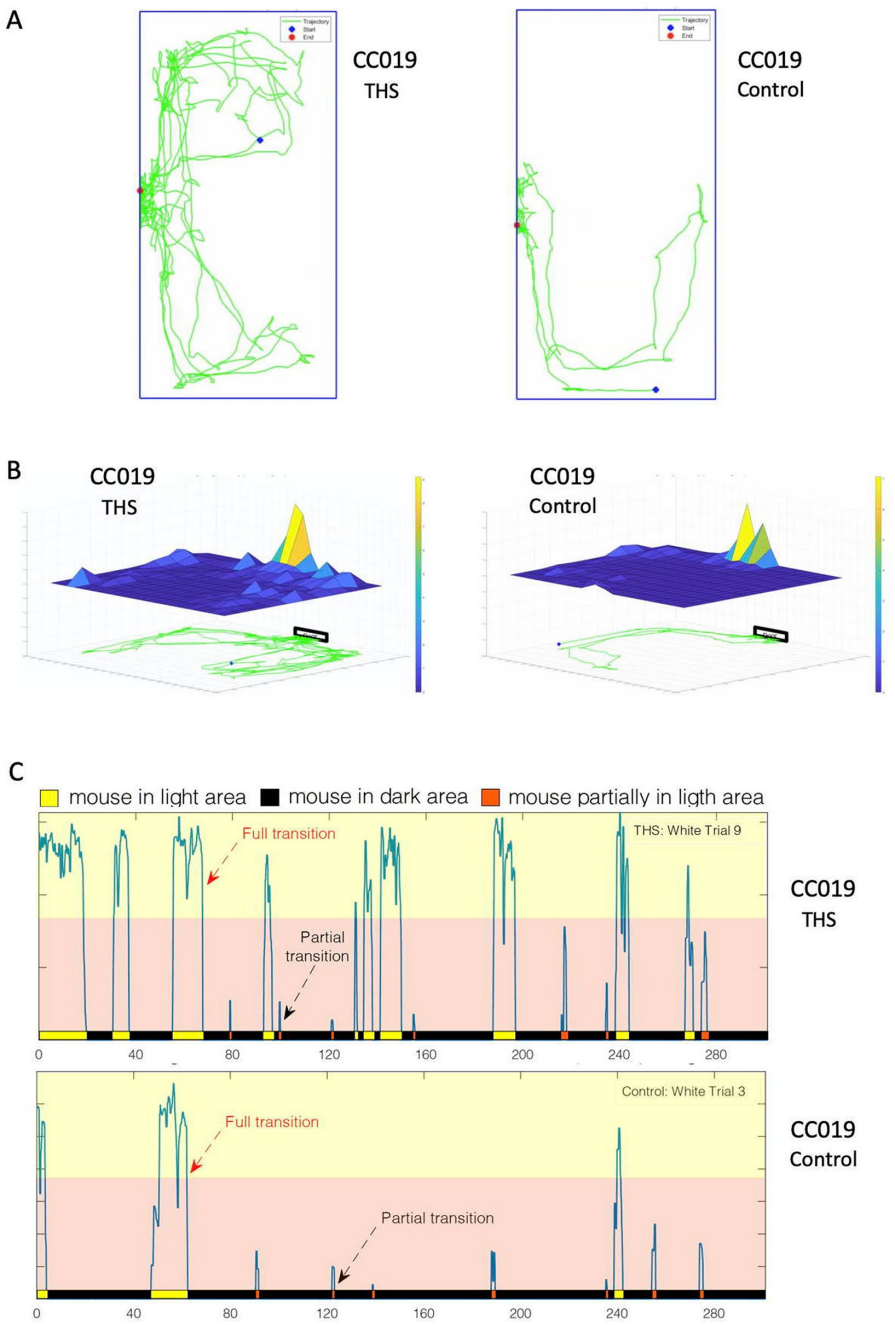
Anxiety test and multivariate phenotyping system. (**A**) Mouse video tracking showing the 5-min trajectory (green line) of a THS exposed (left) and control (right) CC019 mouse in the light compartment of the light–dark box. (**B**) Heatmaps of the THS exposed CC019 mouse (left) or control CC019 mouse (right), showing the cumulative time spent at each location in the light box. (**C**) Representative profile of a THS (top) and control (bottom) exposed CC019 mouse in the light–dark box for the 300 s test duration. Transitions between the light and dark compartments were determined by measuring the relative body area in the light area. The bar at the bottom of the profile indicates the presence of the mouse in the dark area (black), the light area (yellow) or in between the light and dark boxes (orange).

The number and sex of mice used across six CC strains in this study are summarized in Supplementary Table S1. These strains were selected to represent low, intermediate or high anxiety risk tiers in the baseline level study using the light–dark box test^27^. To assess anxiety-like behavior in these mice, each mouse was video-recorded for 5 min one day after 6 weeks of THS or control exposure. A computational pipeline^27^ was deployed to quantify six anxiety-like phenotypes extracted from each video file: latency time to enter the dark box for the first time, number of partial transitions, traveling distance in light, number of full transitions, total time in the light compartment, and average speed in light, where some of these phenotypes, e.g., time spent in light chamber, number of full transitions, and latency to enter the dark box, are frequently used to quantify mouse anxiety levels^35–37^. Representative mouse tracking profiles (Fig. 1A, Supplementary Videos S1 and S2) and the spatial heatmaps of the cumulative time spent in the light compartment (Fig. 1B, Supplementary Videos S3 and S4) are shown for a THS exposed and control exposed CC019 mouse. Figure 1C shows the mouse behavior profiles over time for the same THS exposed CC019 mouse (left) and CC019 control mouse (right).

**Figure 2:**
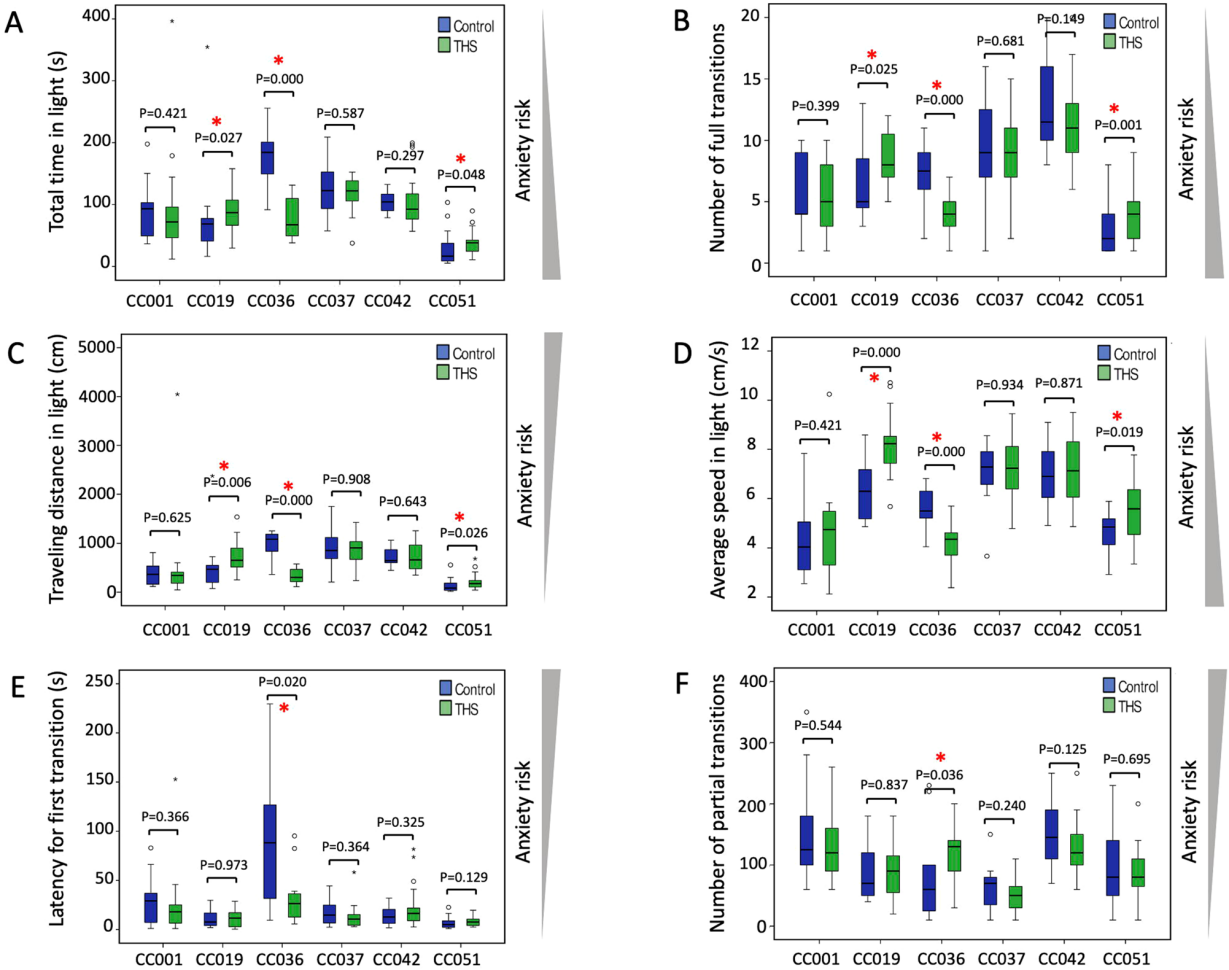
THS exposure effects on anxiety-related phenotypes in six CC strains. The six phenotypes positively or negatively associated with anxiety were measured using the light–dark box. (**A**) Total time in light (s), (**B**) number of full transitions, (**C**) traveling distance in light (cm), (**D**) average speed in light (cm/s), (**E**) latency to first transition (s), (**F**) number of partial transitions. Control mice are shown in blue and THS exposed mice in green. Bars indicate the 1st and 3rd quartiles and the thick horizontal line indicates the median. Error bars indicate minimum and maximum values excluding outliers. *P* values were obtained by Mann–Whitney test. A red asterisk indicates a *P* value < 0.05. The triangle bars on the right side of the plots indicate the direction of anxiety risk. See Table 1 and Supplementary Figure S1 for more details.

We observed significant variations in the baseline levels of six anxiety-like measures between the six CC strains (*P* < 0.05 for all phenotypes), consistent with our previous findings^27^. THS exposure resulted in statistically significant changes in anxiety-like behaviors in the CC019, CC036 and CC051 mice. For instance, compared to control, THS-exposed CC019 mice exhibited significantly higher numbers in the following parameters: total time in the light (*P* = 0.027), number of full transition (*P* = 0.025), traveling distance in light (*P* = 0.006), and average speed in light (*P* < 0.001) (Fig. 2A–D). Similarly, THS-exposed CC051 mice also exhibited increases in these parameters compared to controls (Fig. 2A–D). In contrast, THS exposure caused the opposite effect in CC036 mice, a significant decrease in total time, travelling distance and average speed in light box, latency for first transition and number of full transitions, but a significant increase in number of partial transitions (Fig. 2A–F). To explore the effect of sex on the six anxiety-like measures for each strain, we analyzed male and female mice separately (Supplementary Figure S2), which showed that female mice in each of the three strains (CC019, CC036 and CC051) primarily contributed to the overall significant changes in anxiety-like phenotypes induced by THS exposure (all with *P* < 0.05). No significant difference in these phenotypes was observed in male and female mice between control and THS-treated groups from other CC strains (Supplementary Figure S2).

**Figure 3:**
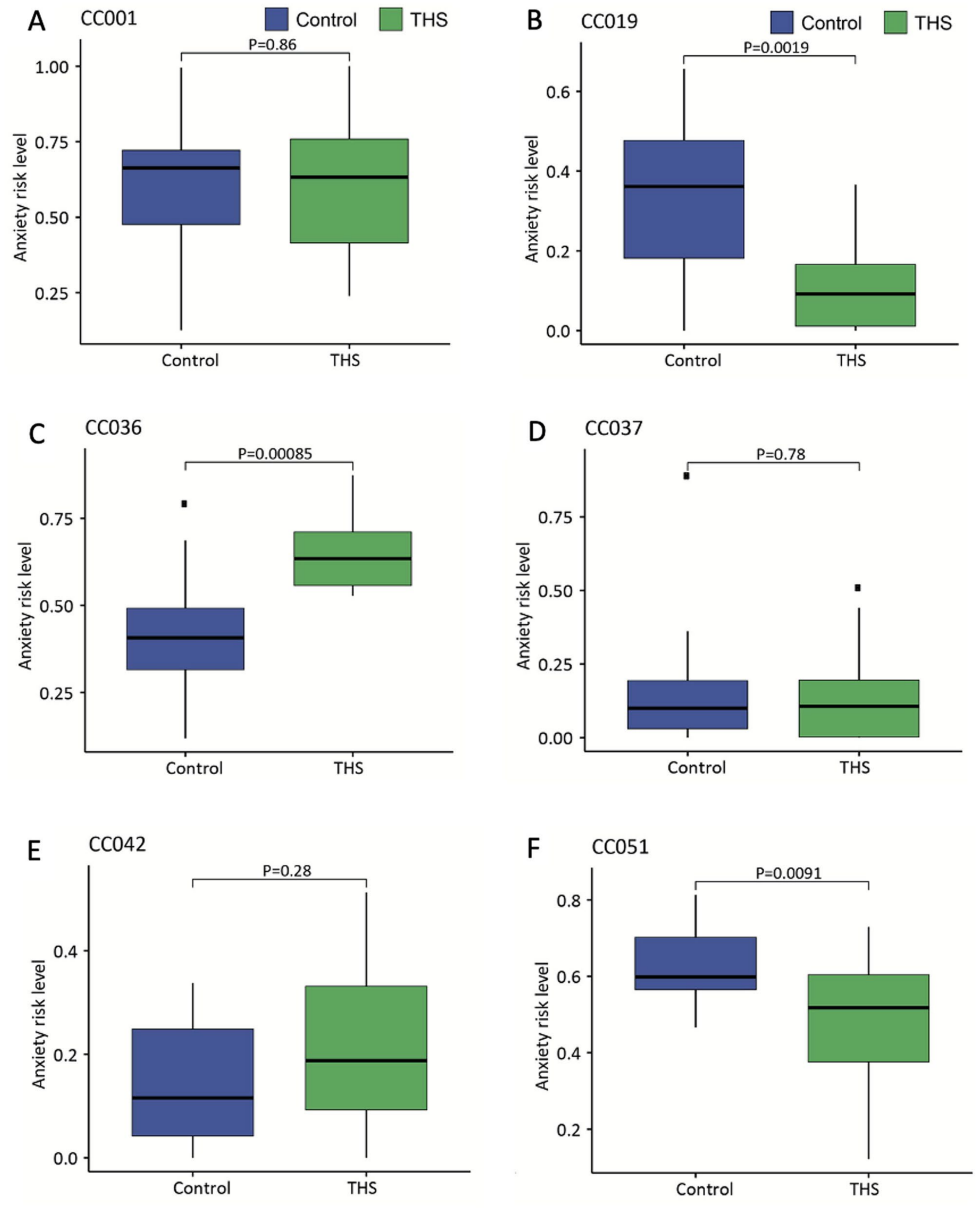
Evaluation of CC mouse anxiety level using the anxiety risk score (ARS). Anxiety risk scores were calculated for control and THS exposed CC001 (**A**), CC019 (**B**), CC036 (**C**), CC037 (**D**), CC042 (**E**) and CC051 (**F**). Bars indicate the 1st and 3rd quartiles and the thick horizontal line indicates the median. Error bars indicate minimum and maximum values excluding outliers. *P* values were obtained by Mann–Whitney test. A red asterisk indicates a *P* value < 0.05.

Finally, to quantitate the effect of THS exposure on anxiety-like behavior, we integrated the six phenotypes into a multivariate anxiety risk scoring model. We first constructed a novel anxiety risk scoring system based on our previously published baseline anxiety data from 445 mice across 30 CC strains using a linear regression model (Table 1, Supplementary Figure S2). We then deployed this multivariate anxiety risk scoring model on the anxiety-like phenotypes measured in our current study to measure the effect of THS exposure on anxiety risk scores (see Supplementary Table S1 for all anxiety-related raw data and corresponding anxiety risk scores). Our results demonstrated that, based on the anxiety risk score (ARS) of the controls, CC042 and CC019 (ARS < 0.2), CC037 and CC036 (0.2 < ARS < 0.6), and CC001 and CC051 (ARS ≥ 0.6) belong to low, intermediate and high anxiety risk tiers, respectively (Fig. 3), consistent with our previous study^27^. THS exposure significantly decreased anxiety risk in CC019 (*P* = 0.002) and CC051 (*P* = 0.009) mice (Fig. 3B,F), whereas THS exposure significantly increased risk of anxiety in CC036 mice (*P* < 0.001, Fig. 3C) in comparison to their corresponding control mice. Taken together, these findings strongly suggest that the effects of THS exposure at young age on anxiety-like behavior are significantly modulated by host genetics and sex, and consequently that THS exposure can lead to opposite effects in different genetic backgrounds.

**Table 1:** Anxiety regression model.

	Estimate	95% CI		*P* value
		Lower	Upper	
(Intercept)	0.503	0.475	0.531	< 0.001
NumberFullTransition	− 0.224	− 0.276	− 0.173	< 0.001
NumberPartialTransition	0.104	0.075	0.133	< 0.001
LatencyTimeFirstTransition	0.080	0.036	0.129	< 0.001
TotalTimeInLight	− 0.242	− 0.328	− 0.155	< 0.001
TravelingDistInLight	0.280	0.190	0.370	< 0.001
AvgSpeed	− 0.270	− 0.325	− 0.214	< 0.001

Estimated coefficients of anxiety-related phenotypes in our anxiety risking scoring model constructed from 445 mice from 30 CC strains, where Intercept is the expected mean anxiety risk score when all anxiety-related phenotypes measured 0.

*CI* confidence interval, *NumberFullTransition* number of full transitions, *NumberPartialTransition* number of partial transitions, *LatencyTimeFirstTransition* latency time to enter the dark box for the first time, *TotalTimeInLight* total time in the light compartment, *TravelingDistInLight* traveling distance in light, *AvgSpeed* average speed in light.

**Figure 4:**
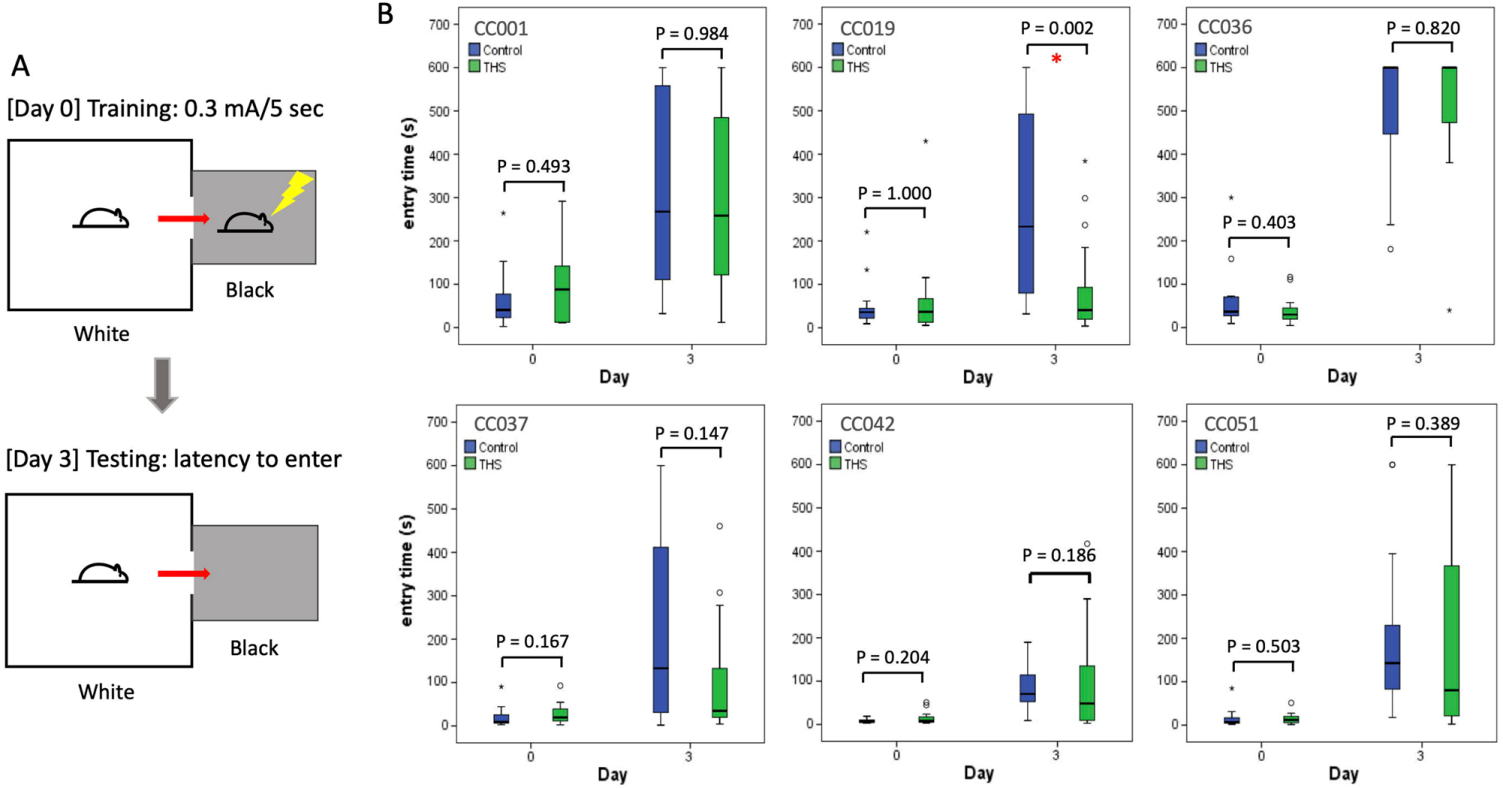
Effects of THS exposure on memory across six CC strains. (**A**) Passive avoidance memory test showing both training and testing phases. During the training phase, the latency to enter the dark compartment is measured. A mild foot shock (0.3 mA for 5 s) is applied. During the testing phase, the latency to enter the dark compartment is measured as an indicator for memory potential. No foot shock is applied during the testing phase. (**B**) The entry time (s) at day 0 and day 3 is shown for all six strains (control mice shown in blue and THS exposed mice in green). Bars indicate the 1st and 3rd quartiles and the thick horizontal line indicates the median. Error bars indicate minimum and maximum values excluding outliers. *P* values were obtained by Mann–Whitney test. A red asterisk indicates a *P* value < 0.05.

To investigate the effect of THS exposure on memory potential in the same cohort of mice, we used the passive avoidance memory test, which measures the delay in time for mice to enter the black box during the training phase (day 0) and testing phase (day 3) as illustrated in Fig. 4A. During the testing phase, 3 days after receiving a mild foot shock, mice with good memory actively avoid entering the dark chamber where they had previously been exposed to the foot shock, whereas mice with poor memory may enter the chamber without much delay. The memory potential was measured 1 day after 6 weeks (from week 4 to 9) of exposure to THS- or control terry cloth. Complete individual data on memory potential are available in Supplementary Table S2.

There was no statistical difference in day 0 entry times between the THS-treated and control mice for all six strains (*P* > 0.05). However, we found that the latency in entry time on the testing day in CC019 mice was significantly shorter after THS exposure as compared with the controls (*P* = 0.002) (Fig. 2B). No significant difference was observed in the other five CC strains between THS exposed and control groups, albeit CC037 showed a trend towards a decrease in memory potential in the THS exposed group (*P* = 0.147). The comparisons between male and female mice in each strain indicated that both THS-exposed male (*P* = 0.0321) and female (*P* = 0.0331) CC019 mice exhibited similar reduction in memory potential on day 3 when analyzed separately. Other strains did not show any sex-related effects on memory. These results suggest that individual genetic background plays an important role in susceptibility to memory disorder caused by THS exposure.

## Discussion

The interplay between genetic background and THS exposure on disease remains unknown due to the lack of relevant animal models. In this work, we investigated the influence of THS exposure on both anxiety and memory in the genetically tractable and diverse CC mouse population-based model system. Mouse model systems offer a distinct advantage for assessing the unknown health risk of THS exposure, as researchers are able to control both the genetic background and environmental components of risk in a well-controlled environment allowing investigations of the effects of THS alone in the absence of SHS exposure, which often confounds human population-based studies of THS exposure. In our study, two cohorts of CC mice representing six CC strains were exposed to THS-laden or control, unexposed, terry cloth samples from 4 to 9 weeks of age. The rationale for treating the mice in a relative early-life window at environmentally relevant doses was based on the presumption that children and young adults are the populations with higher susceptibility to THS exposure effects^1,2^. Our results showing CC strain specific effects of THS exposure indicate that CC mice are a valuable model system for studying the effects of early-life exposure to THS on behavior and for exploring the mechanisms underlying these effects.

Consistent with our previous work^26,27^, significant baseline differences across CC strains for both anxiety-related phenotypes and memory potential were also observed in our current study (Figs. 1, 3, 4). As mentioned before, we chose the six CC strains for the current study based on their anxiety-like phenotype baseline levels without THS exposure. Based on the multivariate anxiety risk scoring system we developed using a linear regression model, these strains were well distributed on the spectrum of the low, intermediate and high anxiety risk tiers. In addition, although we investigated these two categories of aforementioned behavioral phenotypes concurrently on the same mice at the same time after THS exposure, one should treat them as independent behavioral events.

Of the six measures utilized to evaluate the anxiety-like behavior in this study, four measurements including “number of full transitions”, “total time in light”, “traveling distance in light” and “latency for first transition” are well described in the literature. On the other hand, some new measurements were also assessed in this study, such as the “average speed in light” and “number of partial transitions”, which were used to develop a model for calculating anxiety risk score using the light/dark box assay in 445 CC mice across 30 strains in our previous work^27^ and to successfully applied it in current study. More importantly, all six measures (i.e., four traditional and 2 new) have significant and independent contribution to anxiety risk evaluation (Table 1), which confirms the value of the new measures. In any case, this score system may provide a new avenue to assess anxiety in mouse model system.

Anxiety disorders are the most common psychiatric illness in the U.S., affecting nearly 40 million adults (18 and above)^38,39^. Studies suggest that genetic factors are among the best risk factors for anxiety disorders, although anxiety disorders are also influenced by environmental factors^40^. Most recently, we discovered a number of anxiety-related genes in CC strains, which showed significant overlap with human genome-wide association study (GWAS) findings for psychiatric conditions^27^. Many studies have shown connections between cigarette smoking and increased anxiety, including early-life exposure potentially predisposing to increased anxiety symptoms in later life^41^. On the other hand, there have also been reports on the association between cigarette smoking and reduced anxiety in some smokers^42^. Our results across six CC strains are in agreement with such a diverse response*, *i.e., CC019 and CC051 mice displayed reduced anxiety-like behavior, whereas CC036 mice displayed increased anxiety when exposed to THS. Interestingly, further analysis showed that female mice in these groups were the primary driver for these effects. It is well documented that women are more likely to be diagnosed with an anxiety disorder and have a higher prevalence compared to men^43^. The underlying mechanisms for these effects are currently unknown and warrant further investigations. It is generally agreed that most if not all of the psychopharmacological effects of tobacco are due to nicotine^44^, but minor alkaloids, such as anabasine and anatabine have pharmacological activity and may also contribute to the observed phenotypes although they are present in much lower concentrations than nicotine in THS.

For the memory test, almost all mice show increased latency to enter the dark chamber 3 days after receiving a mild foot shock, indicating that memory was formed. The level of memory, however, was variable between different strains, including the untreated mice (Supplementary Table S2), likely due to differences in genetic background between different strains. In addition, our study measured memory potential using the passive-avoidance test, which is based on avoidance of an environment in which mice received a mild foot shock in the training day. We cannot exclude that different strains might have different foot shock sensitivity, which may account for some of the inter-strain differences in observed memory potential. However, this will not influence our conclusion on the effect of THS on memory potential since we compared memory potential between control and THS-treated group of mice from the same strain.

For THS exposure effect on these mice, as shown in Fig. 4, CC019 was the only strain that had significantly reduced memory upon THS exposure (*P* = 0.002). It should be noted that this strain has been found to display a high incidence of spontaneous congenital hydrocephalus (http://csbio.unc.edu/CCstatus/index.py?run=availableLines). We screened the CC019 mice for this disorder and excluded symptomatic mice prior to assignment to this study. Notably, a link has been established between congenital hydrocephalus and a variety of cognitive disorders including memory and behavioral problems, especially in young people^45^. As with CC019 mice, even though those tested were not manifesting the symptoms of hydrocephalus at testing, their genetic background may predispose them to environmental exposure-induced cognitive decline. Therefore, our observation that CC019 mice were more sensitive to THS exposure effects compared to other strains may not be unexpected.

Our original objective was to show whether the phenotypic changes in behavior could prove the hypothesis that host genetic background affects THS-induced behavioral alterations. However, the current study only included six CC strains which does not afford us sufficient statistical power for GWAS-based research. Based on published power simulations, varying the number of CC strains, the number of replicates for each strain and the QTL effect size, it is estimated that 30 strains with at least five replicates and an effect size of 30–40% allows 80% of power^46^. Based on our results, future studies are warranted by increasing the number of strains to identify the detailed genetic differences between the six strains, i.e., which genetic loci or genes are strongly associated with THS-induced changes in memory and anxiety. In our study we focused on assessing fear conditioned memory. Fear conditioning depends on signaling in the amygdala. Future studies of THS exposure effects using memory assays that test spatial memory or recognition memory that depend on hippocampal signaling should also be conducted. Ideally, a combination of different types of assays may result in a more comprehensive assessment of the behavioral changes associated with THS exposure. Finally, in our study, the levels of nicotine and/or cotinine were not assessed across CC strains due to detection limitations using LC–MS/MS procedure as described previously^10,11^. It is not unlikely that the differences in behavioral phenotypes observed across different CC strains are related to strain specific differences in absorption and metabolism of specific THS compounds.

## Conclusions

Despite substantial progress in identifying the health impacts of THS exposure, it is still largely unclear how THS exposure interacts with host genetic background affecting behavior. In general, the pathogenesis of behavioral disorders is a complex process involving intricate interactions between genetic variants, environmental influences (exposures, lifestyle differences, etc.), sex and psychological mechanisms. In this study, we demonstrated strain-dependent effect of THS exposure on both anxiety-like behavior and memory and highlighted the importance of genetic background in modulating THS exposure effects. The experiments described here warrant further investigation using a larger number of CC strains to identify the genes controlling these responses and using cross-species genetic analysis to translate our mouse findings into human study and uncovering the molecular mechanisms of THS exposure related disease.

## Supplementary information

Supplementary Information 1.

Supplementary Information 2.

Supplementary Information 3.

Supplementary Video 1.

Supplementary Video 2.

Supplementary Video 3.

Supplementary Video 4.

## Data Availability

All data generated or analyzed during this study are included in this published article and its Supplementary Information files.
