# Supplementary material for: Genetic background influences the effect of thirdhand smoke exposure on anxiety and memory in Collaborative Cross mice
Source: Sci Rep. 2021 Jun 24;11:13285. doi: 10.1038/s41598-021-92702-1 (PMC8225773; doi:10.1038/s41598-021-92702-1)
Supplement: Supplementary file 1 — Supplementary Information 1. [file 41598_2021_92702_MOESM1_ESM.docx]

**Supplementary Information**

**Genetic Background Influences the Effect of Thirdhand Smoke Exposure on Anxiety and Memory in Collaborative Cross Mice**

Li He, Pin Wang, Suzyann F. Schick, Abel Huang, Peyton Jacob III, Xu Yang, Yankai Xia, Antoine M. Snijders, Jian-Hua Mao, Hang Chang and Bo Hang

**Supplementary Figures**

**Supplementary Figure S1.**


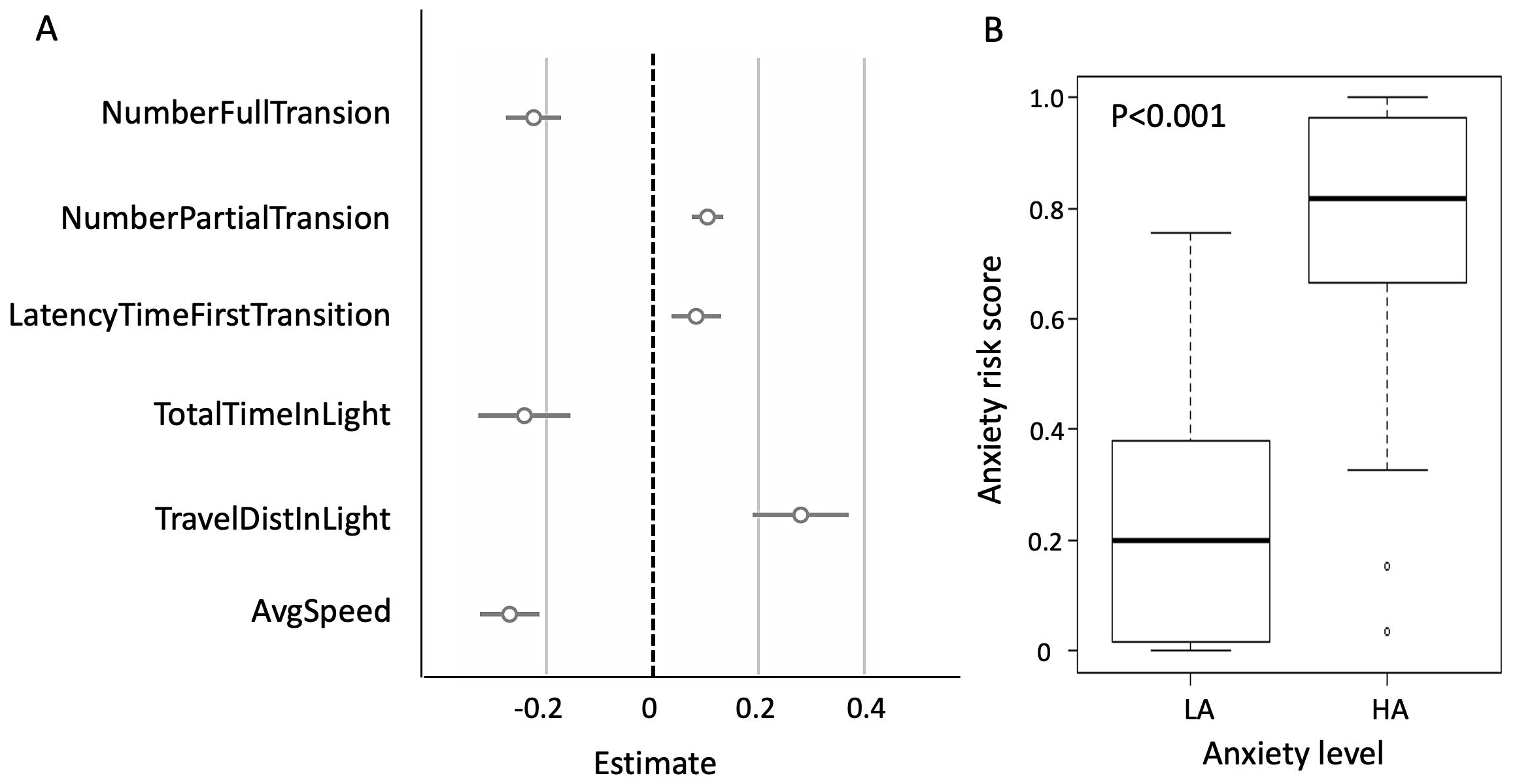


**Anxiety risk scoring model and the corresponding anxiety risk score evaluated on ground-truth cohort.** **A**. Estimated coefficients of six anxiety-related phenotypes in the anxiety risk scoring model. NumberFullTransition: number of full transitions; NumberPartialTransition: number of partial transitions; LatencyTimeFirstTransition: latency time to enter the dark box for the first time; TotalTimeInLight: total time in the light compartment; TravelingDistInLight: traveling distance in light; AvgSpeed: average speed in light. **B**. Anxiety risk score evaluated with the anxiety risk scoring model on 445 mice from 30 CC strains within low anxiety (LA) and high anxiety (HA) groups, as discovered in our previous study^27^.

**Supplementary Figure S2.**

**Evaluation of anxiety risk levels in male and female CC mice.** Anxiety risk scores were calculated for control and THS exposed six CC strains. Bars indicate the 1^st^ and 3^rd^ quartiles and the thick horizontal line indicates the median. Error bars indicate minimum and maximum values excluding outliers. P-values were obtained by Mann-Whitney test. A red asterisk indicates a P-value < 0.05. M: male; F: female.

**Supplementary Videos:**

**Supplementary Video S1:** Behavioral video tracking of a CC019 mouse after THS exposure in the light/dark box assay (5 min).

**Supplementary Video S2:** Behavioral video tracking of a control exposed CC019 mouse in the light/dark box assay (5 min).

**Supplementary Video S3:** Heatmap of a CC019 mouse after THS exposure in the light/dark box assay (5 min).

**Supplementary Video S4:** Heatmap of a control exposed CC019 mouse in the light/dark box assay (5 min).

**Supplementary Tables:**

**Supplementary Table S1:** Anxiety raw data (six anxiety-related phenotypes) and corresponding anxiety risk scores (ARS).

See the Excel document.

**Supplementary Table S2:** Raw data (entry times for day 0 and day 3) from memory test and statistical analysis.

See the Excel document.
